# Healthy eating and obesity prevention for preschoolers: a randomised controlled trial

**DOI:** 10.1186/1471-2458-10-220

**Published:** 2010-04-28

**Authors:** Helen Skouteris, Marita McCabe, Boyd Swinburn, Briony Hill

**Affiliations:** 1 School of Psychology, Faculty of Health, Medicine, Nursing and Behavioural Sciences, Deakin University, Melbourne, Australia; 2 School of Exercise and Nutrition Sciences, Faculty of Health, Medicine, Nursing and Behavioural Sciences, Deakin University, Melbourne, Australia

## Abstract

**Background:**

Developing effective prevention and intervention programs for the formative preschool years is seen as an essential step in combating the obesity epidemic across the lifespan. The overall goal of the current project is to measure the effectiveness of a healthy eating and childhood obesity prevention intervention, the MEND (Mind Exercise Nutrition Do It!) program that is delivered to parents of children aged 2-4 years.

**Methods/Design:**

This randomised controlled trial will be conducted with 200 parents and their 2-4 year old children who attend the MEND 2-4 program in metropolitan and regional Victoria. Parent-child dyads will attend ten 90-minute group workshops. These workshops focus on general nutrition, as well as physical activity and behaviours. They are typically held at community or maternal and child health centres and run by a MEND 2-4 trained program leader. Child eating habits, physical activity levels and parental behaviours and cognitions pertaining to nutrition and physical activity will be assessed at baseline, the end of the intervention, and at 6 and 12 months post the intervention. Informed consent will be obtained from all parents, who will then be randomly allocated to the intervention or wait-list control group.

**Discussion:**

Our study is the first RCT of a healthy eating and childhood obesity prevention intervention targeted specifically to Australian parents and their preschool children aged 2-4 years. It responds to the call by experts in the area of childhood obesity and child health that prevention of overweight in the formative preschool years should focus on parents, given that parental beliefs, attitudes, perceptions and behaviours appear to impact significantly on the development of early overweight. This is 'solution-oriented' rather than 'problem-oriented' research, with its focus being on prevention rather than intervention. If this is a positive trial, the MEND2-4 program can be implemented as a national program.

**Trial Registration:**

Australian New Zealand Clinical Trials Registry ACTRN12610000200088

## Background

Epidemiological studies indicate that childhood overweight is a serious problem in Australia, with an estimated 1 in 4 Australian school aged children and adolescents either overweight or obese [1]. Alarmingly, obesity is seen in even younger children, with rates of overweight and obesity almost doubling in a sample of 114,669 Australian 4-year-olds over the period 1995-2002 [2]. More recently, data from 4934 4- to 5-year-olds from the first wave of the nationally representative Longitudinal Study of Australian Children revealed that 15.3% of preschoolers were overweight and a further 5.2% obese [3]. Thus, the cost to society of obesity is high, with Access Economics estimating that the total annual cost of obesity to Australian society in 2008 was $58.2 billion, up from $21 billion in 2005 [4]. Furthermore, obesity in adults is strongly associated with the development of cardiovascular disease, type 2 diabetes mellitus and some cancers [5]. Overweight and obese children and adolescents are also at risk of a range of health problems and have an increased risk of premature death in adulthood; there are both immediate complications and long term consequences of child and adolescent obesity [6-8]. Research has shown that obesity can also have psychological consequences among children, such as increased stigmatization and depression [9,10]. The desire for thinness is present in girls as young as six years of age [11] and our research reveals that 4-year-old children are already concerned about their hair and clothes [12]. Higher weight status in 5-year-old girls has also been found to be associated with lower self-concept [13]; hence, preschoolers are at risk of obesity's negative psychological consequences.

Whilst strong evidence supports the role of non-modifiable genetic factors in energy storage patterns [14], these are insufficient to explain the increase in childhood overweight in recent years. Given that child weight is a multi-determined condition, researchers now argue that obesity prevention/intervention strategies must influence multiple determinants of child risk factors for overweight and obesity (i.e., increase in high-calorie, nutrient-poor food consumption, increase in sedentary behaviours, and a lack of physical activity, as well as parental attitudes and behaviours) [15,16]. The primary institution influencing preschool children is the family; consequently, many of the potentially modifiable determinants of risk factors for overweight and obesity in the preschool years will have roots within the family context [17]. Indeed, positive associations between certain aspects of parenting and preschool childhood overweight/obesity have been documented: parenting practices/behaviours and the beliefs supporting these strategies including maternal feeding practices, such as restricting access to foods and pressuring to eat [18-22]; reduced parental instrumental support for engagement in health promoting behaviours (e.g., not providing access or prompts for healthy eating or physical activity) [16,23]; low levels of parental modelling of healthy eating habits and physical activity [16,24,25]; and low parental nutritional knowledge [26].

In recent years, researchers have also examined the ways in which parents "*parent*" their children and found that particular parenting and feeding styles (i.e., authoritarian and permissive feeding styles) impact on young children's weight status [3,16,27,28]. Yet, despite widespread evidence that parenting behaviours, beliefs, and knowledge as well as parenting and feeding styles, are important in the development and maintenance of child obesity, relatively few studies have examined systematically whether targeting parental influences to modify these risk factors within a complex intervention will have an effect on weight status in preschool children.

Our systematic review (Skouteris H, McCabe M, Swinburn B, Newgreen V, Sacher P, Chadwick P: Parental influence and obesity prevention in preschoolers: A systematic review of interventions, submitted) revealed that worldwide only eight RCTs have been conducted since 1995 on prevention of obesity in the 2-4 year old age group, and only one of these was conducted in Australia [29]; the age range targeted in this Australian study was 3-10 years, and the four-by-two-hour weekly group parent-education intervention program was for overweight children with a 3-month follow up period. This brief intervention for parents was effective in reducing childhood overweight at 3 months follow-up. Other studies have also shown that teaching parents about nutrition and healthy lifestyle behaviours results in improved knowledge [30] and health behaviours [15] and this can result in weight improvements for their children [31]. A key conclusion in the literature is the lack of translation of knowledge to action [32,33] and for this reason, future interventions should also specifically target strategies and techniques which parents can use to modify their child's dietary and physical activity patterns. It is argued that interventions that involve parents in a significant way may be particularly effective; they are likely to work by improving parental engagement, skills, and ability to provide opportunities for active play for their child and to create a healthy nutritional home environment [34].

The MEND (Mind, Exercise, Nutrition, Do It!) 2-4 program was designed to address this need for a healthy lifestyle program in the early years of childhood as well as a secondary obesity prevention initiative. MEND 2-4 is a 10-week community-based, multi-component healthy lifestyle program that is offered free to families with young children aged 2-4 years, irrespective of weight. The program aims to encourage healthy habits around diet and activity from an early age. It is a highly structured group program allowing parents to be supported in establishing healthy habits and making healthy behaviour changes at their own pace [35]. MEND 2-4 leaders are provided with comprehensive training by MEND Australia, and receive a leader's manual together with a full resource kit. The MEND 2-4 program is open to parents of children of any weight, although it is recommended that 'at risk' children be targeted (via referrals from General Practioners, Pediatricians, or Maternal and Child Health Nurses), including children who have/are: parental obesity, inactive and/or have poor eating habits, a risk of developing co-morbidities (e.g., family history of cardiovascular disease, type 2 diabetes, specific ethnic minorities). The overall goal of the current project is to measure the effectiveness of the MEND 2-4 program by conducting a randomised controlled trial (RCT).

## Methods/Design

### Overall study design

**Figure 1:**
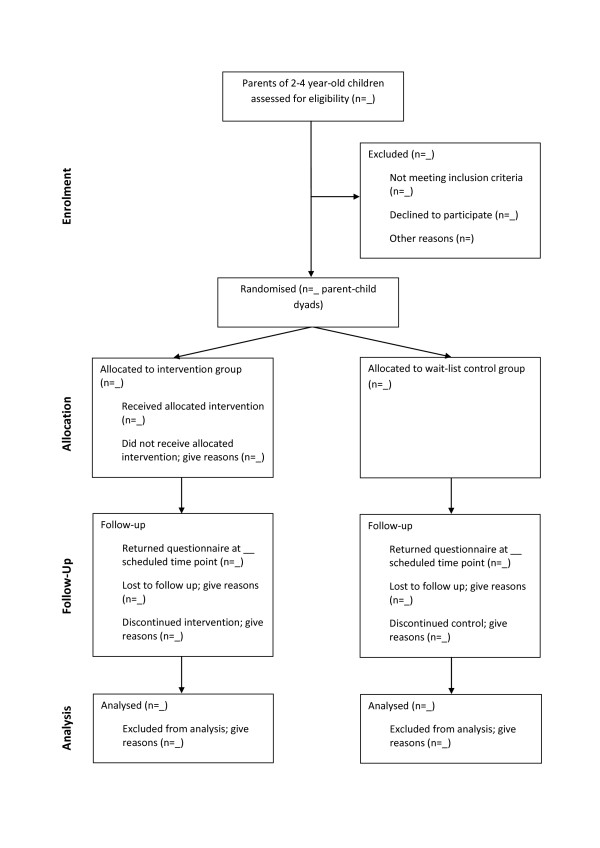
**Study Design**. (attached as separate document).

This study is a randomised controlled trial with parents of 2- to 4-year-olds randomly allocated to either the intervention or control groups and will be conducted and reported in line with CONSORT recommendations [36] (see Figure 1). Baseline assessment of study variables will occur at recruitment. Follow up assessments will take place at the end of the intervention (10 weeks post baseline measures) and at 6 and 12 months post intervention. This study was approved by the Deakin University Human Research Ethics Committee on the 7^th ^December 2009 (DU-HREC 2009-180).

### Hypotheses

In comparison to the wait-list control group, we predict that at the completion of the 10-week intervention program, and at 6 and 12 months post intervention, the intervention group preschoolers will: (1) demonstrate greater consumption of fruit and vegetables, and a decrease in consumption of high fat, high sugar cordials, soft-drinks and juices and non-essential, energy dense snack foods; (2) exhibit less problematic eating habits, specifically higher responsiveness to satiety, less fussiness, and less neophobia; (3) demonstrate greater increases in physical activity and greater decreases in time spent in sedentary behaviours, specifically TV viewing; (4) exhibit lower BMI-for-age z scores.

In comparison to the wait-list control group, we hypothesise at the completion of the intervention program, and at 6 and 12 months post intervention, the intervention group parents will: (1) increase the frequency with which they limit the offering of high fat, high sugar cordials, soft-drinks and juices and non-essential, energy dense snack foods to their child; (2) increase the frequency with which they offer vegetables and fruit, water and milk (rather than cordials, soft-drinks and juices) to their child; (3) demonstrate improved knowledge regarding key aspects of child eating, physical activity and sedentary behaviours relevant to the prevention of obesity and improving child health generally; (4) reduce the frequency with which they use restrictive feeding practices and increase the frequency with which they use covert methods of controlling access to unhealthy foods; (5) increase the frequency with which they model healthy behaviours around diet and physical activity; (6) provide greater opportunities for children to engage in physical activity and reduce their opportunities for engagement in sedentary behaviours.

### Participants

The participants will be 200 parents of 2-4 year old children. Parents will be eligible to take part if they can provide informed consent, are 18 years of age or older, and can read and write English. After assessment, parents who meet the inclusion criterion, and do not display exclusion criteria, will be randomly allocated to either the MEND 2-4 intervention (*n *= 100) or the wait-list control group (*n *= 100). A coded, double-blinded, variable-length permuted blocks randomised treatment allocation schedule produced by computer algorithm will be used.

### Recruitment strategies

Parent-child dyads will be recruited through local newspaper and kindergarten advertisements, maternal and child health centres, and general practice medical centres. Every effort will be made to include families from diverse ethnic backgrounds, given that different SES and ethnic groups exhibit differences in parental attitudes and beliefs in relation to feeding practices, child weight gain, and physical activity [37].

#### Procedure

##### The Intervention

The MEND 2-4 program involves 10 weekly 90 minute workshops relating to general nutrition, physical activity and behaviours, that are typically held at community health and maternal and child health centres, where parents and their preschool-aged children attend together. Each program group will consist of 6-10 child-parent dyads and a MEND 2-4 trained program leader. Program leaders, who are trained extensively by MEND Australia prior to starting with a group, will be monitored and evaluated by MEND Australia staff to ensure their practice is in accordance with MEND 2-4 guidelines; parental feedback on program leaders will also be obtained. Each session involves 30 minutes of guided active play, where parents can learn how to play with their children; 15 minutes of healthy snack time based on an evidence-based exposure-based technique to promote acceptance and increased intake of fruit and vegetables [38] and 45 minutes where the children participate in supervised crèche-style, creative play activities and concurrently the parents attend an interactive education and skill development session, based on evidence-based group-based parent-training principles [39]. Table 1 outlines the weekly education topics, and parents will receive handouts on these topics weekly. Pilot data reveals low attrition and that parents value the program and attend all 10 sessions. In the initial MEND 2-4 trials (UK) the participation rate was 87.6% which is high for developmental research [35].

**Table 1:** Discussion topics and intervention content of MEND 2-4 program

Week	Workshop Title	Discussion Topic(s)	Intervention Content
1	**Welcome & introduction**	Meet the leader and pre-program measurements	Meet and welcome the families to their first MEND 2-4 physical activity and snack-time session; parents complete all pre-program forms and questionnaires and accurate child and parent anthropometric measurements (height and weight) taken.

2	**Introduction**	Introductions and individual expectations	Discussion of individual expectations and introduction of MEND 2-4 program and practicalities; Introduction of parents/carers to the behavioural model of parenting (customised as the 4Cs model (Causes, Consequences, Consistency and Copying) for the purposes of MEND 2-4).

3	**Healthy eating for families**	Healthy eating	Introduction to the five food groups, visual samples of excess sugar and fat found in foods. Discussion of appropriate treats and rewards and toddler intake of drinks.

4	**Be healthy, get active!**	Non-TV activities for toddlers	Importance of limiting TV watching among toddlers; Goal setting activity towards achievement of MEND 2-4 TV time guidelines (maximum of 1-2 hours of TV per day). Discussion of active play and activity ideas to replace time spent previously watching TV.

5	**What's in your child's food?**	Reading food labels	Reading food labels; MEND 2-4 label reading guidelines and identification of MEND 2-4 friendly food.

6	**Food without fuss**	Dealing with fussy eaters	Normalisation of fussy eating and common causes; importance of consistency around mealtimes and ideas for managing fussy eating behaviour. Strategies to reduce fear and unhelpful parenting behaviour around food at mealtimes.

7	**Portion sizes**	Healthy eating and portion sizes	Introduction to the MEND 2-4 portion sizes with visual demonstration; demonstration of difference between toddler and adult portion sizes.

8	**Fun with food**	Cooking together	Demonstration of ideas for making food preparation fun and including fruits and vegetables, how to actively involve children in preparation of snacks and parents/carers and child having fun with food together.

9	**Encouraging healthy habits**	Rules, routines and tantrum management	Establishing health as a priority within the family life-cycle. Helpful strategies for dealing with behaviours that may be resistant to change. MEND 2-4 sleep guidelines for toddlers.

10	**Farewell and graduation**	Evaluation and measurement	Collection of post-program measurements; MEND 2-4 certificates provided; information about follow-up activities or other local groups they may like to attend at the end of the MEND 2-4 Program.

##### Control Group

Parents in this group will be assessed (same measures as intervention group) at baseline and will have follow-up assessments at the same time as the intervention group (10 weeks, 6 months and 12 months post baseline assessment). These parents will be offered the intervention at 15 months post baseline assessment.

##### Measures

At recruitment, parents will be asked to complete an informed consent form confirming their participation in the study. All of the following measures will be administered at baseline, the end of the intervention, and at 6 and 12 months post intervention. The Demographic Questionnaire and Food Frequency Questionnaire for adults are the only exceptions, in that they will be administered at baseline and at 12 months post intervention only.

#### Primary outcome

##### Child eating habits and daily dietary intake

The Children's Eating Behaviour Questionnaire (CEBQ) will be used to assess child eating styles/habits, specifically, fussiness and responsiveness to satiety [40]. Typical food intake for children and their parents will be assessed using a semi-quantitative food frequency questionnaire [41] developed for Australian preschool children. The Eating and Physical Activity Questionnaire (EPAQ) is a two page document which asks specifically about foods which have been associated with obesity in the literature - particularly beverages, fruits and vegetables, processed snack foods and takeaway foods. Quantities are in general household measures and refer to either the previous day or typical intake. Standard serving sizes are based on the Australian Guide to Healthy Eating [42]. The EPAQ has been assessed against a 24-hour dietary recall in 90 parents of preschoolers and found to be valid [41]. Reliability has been found to be adequate [43]. This method of food recall has low participant burden and hence is both time and cost-efficient [44].

#### Secondary outcomes

##### Duration of daily child physical and sedentary activities

The Physical Activity Questionnaire for Preschool-aged Children (Pre-PAQ^®^) is a parent self-report measure that provides a list of 24 different types of physical and sedentary activities and requires parents to report on the time spent in the activities their child did "yesterday" and "last weekend" [45]. Parental proxy-reporting of their children's time-use has been used widely in developmental research [46-50], and has been shown to be a valid method of assessing individual differences in young children's time-use habits [51].

##### Parental knowledge of nutrition

Knowledge of child nutrition will be assessed with subscales of the Nutrition Knowledge Questionnaire [52].

##### Anthropometry

Height and weight will be measured with a stadiometer and standardised digital scales, respectively, at all assessments for children and at baseline, and at the 6 and 12 months follow up assessment time points for parents. Children and parents will be weighed each time wearing light clothing and no shoes. Height and weight measures will then be converted to a Body Mass Index (BMI, kg/m^2^) for each participant. Body Mass Index will be standardized for age and gender using BMI-for-age z-scores and change will be assessed using BMI z score slope [19] following WHO recommendations for children this age [53].

##### Parental behaviours and cognitions pertaining to feeding, eating and physical activity

**(1) *Maternal Child Feeding Patterns*: **The Preschool Child Feeding Questionnaire (PCFQ) [54] will measure mother's anxiety about feeding, responsibility for feeding, pressure to eat, and controlling feeding, and her concerns for her child's weight. The Child Feeding Questionnaire (CFQ) [55,56] will assess maternal restriction of access to food and monitoring of food. **(2) *Parental Eating*: **Typical daily food intake for parents will be assessed using the National Nutrition Survey Food Frequency Questionnaire [57]. **(3) *Parental modelling of physical and sedentary activities*: **The Active Australia Survey [58] (a validated questionnaire that measures physical activity behaviour in adults) will be used to measure parental engagement in physical and sedentary activities (duration and nature); this is retrospectively reported by parents for the last seven days. **(4) *Parental encouragement*: **Parents will also be asked to rate on a 5-point likert scale (1 = not at all encouraging; 5 = extremely encouraging) how encouraging they feel they are of their child participating in physical activities, eating fruits and vegetables, reading books, and watching TV; similarly, they will be asked to rate how discouraging they are of their child participating in sedentary activities, eating energy-dense snack foods and watching TV. **(5) *Parent cognitions*: **Parents will be asked to rate on a 5-point likert scale (1= not at all important; 5 = extremely important) how important it is to them that they are, and their child is, involved in sporting/physical activities, spends time reading, eats fruits and vegetables, socializes with other children, plays outdoors, plays indoors, and watches TV/DVDs, as well as how approving they both are of the television programs and videos their child views. **(6) *Parental concern for child weight*: **The concern for child's weight will be measured in the PCFQ [54]. **(7) *Child food neophobia*: **Pliner's Child Neophobia Scale [59] will be used to determine the extent of a child's reluctance to eat unfamiliar food.

### Qualitative interviews

We will invite 20 parents from the intervention to take part in an interview to obtain qualitative data in relation to barriers to creating a healthy home environment and the support or resources that parents find most useful in establishing and/or maintaining healthy behaviours and attitudes around diet and physical activity for themselves and their preschool child(ren). These interviews will take place 12 months post the intervention, and will inform us on the strengths of the intervention and subsequent program development.

### Power, sample size, and retention

The primary outcome for this intervention is child eating habits and dietary intake. Both the expected clinical effect of the intervention and variability of the primary outcome measure are important considerations. Thus, sample size calculations were based on Australian data by Mathews et al. that provide relevant parameter estimates from three studies in the Barwon-South Western region of Victoria (*n *= 950) with children aged 2-4 years of age [60]. Vegetable consumption will require the largest sample size to demonstrate change over time, compared to other dietary outcomes, such as sweetened drinks, packaged snacks (e.g., chips, muesli bar), confectionary/chocolate, cake/sweet biscuits, and fruit, based on sample size analyses. As there are no quantitative dietary recommendations for children less than 4 years old in Australia, we adopted Campbell et al.'s suggestion of a 25% increase in vegetable consumption as a minimum target [61]. Based on Mathews et al.'s finding that 63% of pre-school children had ≤1 serve of vegetables per day [60], we assume that the intervention will have a 25% absolute effect, that is the proportion of children in the intervention group who only have ≤1 serve of vegetables per day will change from 63% to 43%. We calculated the sample size necessary to detect a clinically meaningful difference in vegetable consumption, significant at the 0.05 level, with a power of 0.8; this calculation resulted in a sample of 100 parents in each group. Making a prudent allowance for attrition of 20% (attrition in a large longitudinal study with multiple time points over a 3-year period by Clark et al. [62]) the adjusted number per treatment condition is 120. A total of 240 parents of 2-4 year old children will therefore be recruited at baseline to allow for a final sample of 200. The final sample size of 200 participants is also sufficient to detect medium effect sizes in the secondary measures with a power of 0.8 at α = 0.05 [63]. Every CI on this research team has had experience in retention of participants in longitudinal studies. To facilitate retention we will build rapport with the participants by a regular newsletter and advance notification of assessments. To increase retention rates the CIs have found two measures are very helpful: 1) Parents who remain in the study will be entered into prize draws for gift vouchers. The amount needs to include a large grand prize plus multiple minor prizes. Prizes will be drawn each year. 2) Birthday cards will be sent out to each child participant.

### Analyses

We will adhere to 'intention-to-treat' principles following CONSORT Statement guidelines to prevent introduction of systematic bias [34]. As such, baseline data will be secured prior to treatment allocation, missing values will be scrutinized to check for non-random distribution and primary analyses will be executed twice: once using observed data, and once using multiple imputation under multivariate normal assumptions using methods given by Schafer [64], so that all participants will be analysed in their allocated condition. Analyses of covariance (with "other relevant variables" from above as covariates) will test the between group differences in the primary and secondary outcomes at each assessment time point and across time points for the parents and children. Finally, the interview transcripts will be analysed using elements of phenomenology and thematic content analysis [65], as demonstrated by Clark et al. and Powell et al. [66,67].

## Discussion

Obesity has reached epidemic proportions in Australia and calls have been made for strategies to reverse this unhealthy trend [68,69]. Early prevention of obesity, that is, developing effective prevention and intervention programs for the formative preschool years, is seen as one essential step in combating the obesity epidemic across the lifespan [70]. The preschool years have been identified as a critical period in childhood for the development of childhood obesity [71] because the eating and physical activity habits that contribute to later obesity become established during these formative years [72,73]. Targeting preschool aged children is central to preventing obesity, because development at this life stage is more malleable than it is in later childhood and adolescence [47], and risk factors of overweight can be more easily modified [74]. Despite these findings, there is a paucity of strategies aimed directly at preschool children [70,75]. This study aims to address this urgent need to develop and test the effectiveness of prevention programs for children at the preschool age. Such prevention programs need to be family-based [76], because the primary social force that influences young children's health behaviour and development is the parent [17,37]. Our intervention will provide information to parents and support them to establish and/or maintain healthy behaviours and attitudes around diet and physical activity for themselves and their preschool child(ren). If effective, this program could protect children from the development of obesity and its associated psychological, social and economic costs. Furthermore, this study has the potential to strengthen our knowledge base of health-promoting strategies that can be aimed at parents, which will hopefully lead to future national and international policy changes in relation to obesity prevention as well as the promotion of change at a global level.

## Competing interests

The authors declare that they have no competing interests.

## Authors' contributions

Together, HS, MM, and BS wrote and designed the study subsequently funded by the Australian Research Council, and modified it for publication. BH is the research assistant appointed to manage the collection of data. All authors read and approved the final manuscript.

## Pre-publication history

The pre-publication history for this paper can be accessed here:

http://www.biomedcentral.com/1471-2458/10/220/prepub
